# Glutamine attenuates the inhibitory effect of methotrexate on TLR signaling during intestinal chemotherapy-induced mucositis in a rat

**DOI:** 10.1186/1743-7075-11-17

**Published:** 2014-04-17

**Authors:** Igor Sukhotnik, Yulia Pollak, Arnold G Coran, Janna Pilatov, Jacob Bejar, Jorge G Mogilner, Drora Berkowitz

**Affiliations:** 1 The Bruce Rappaport Faculty of Medicine, Technion-Israel Institute of Technology, Laboratory of intestinal adaptation and recovery, Haifa, Israel; 2 Department of Pediatric Surgery, Bnai Zion Medical Center, 47 Golomb St., P.O.B. 4940, Haifa 31048, Israel; 3 Pathology, Bnai Zion Medical Center, Haifa, Israel; 4 Gastroenterology, Bnai Zion Medical Center, Haifa, Israel; 5 Section of Pediatric Surgery C.S. Mott Children’s Hospital and University of Michigan Medical School, Ann Arbor, MI, USA

**Keywords:** Toll-like receptor 4, Methotrexate, Mucositis, Glutamine

## Abstract

Toll-like receptor 4 (TLR-4) is crucial in maintaining intestinal epithelial homeostasis, participates in a vigorous signaling process and heightens inflammatory cytokine output. The objective of this study was to determine the effects of glutamine (GLN) on TLR-4 signaling in intestinal mucosa during methotrexate (MTX)-induced mucositis in a rat. Male Sprague–Dawley rats were randomly assigned to one of four experimental groups of 8 rats each: 1) control rats; 2) CONTR-GLN animals were treated with oral glutamine given in drinking water (2%) 48 hours before and 72 hours following vehicle injection; 3) MTX-rats were treated with a single IP injection of MTX (20 mg/kg); and 4) MTX-GLN rats were pre-treated with oral glutamine similar to group B, 48 hours before and 72 hours after MTX injection. Intestinal mucosal damage, mucosal structural changes, enterocyte proliferation and enterocyte apoptosis were determined 72 hours following MTX injection. The expression of TLR-4, MyD88 and TRAF6 in the intestinal mucosa was determined using real time PCR, Western blot and immunohistochemistry. MTX-GLN rats demonstrated a greater jejunal and ileal mucosal weight and mucosal DNA, greater villus height in ileum and crypt depth and index of proliferation in jejunum and ileum, compared to MTX animals. The expression of TLR-4 and MyD88 mRNA and protein in the mucosa was significantly lower in MTX rats versus controls animals. The administration of GLN increased significantly the expression of TLR-4 and MyD88 (vs the MTX group). In conclusion, treatment with glutamine was associated with up-regulation of TLR-4 and MyD88 expression and a concomitant decrease in intestinal mucosal injury caused by MTX-induced mucositis in a rat.

## Introduction

The family of mammalian Toll-like receptors (TLRs) are homologs of the *Drosophila* Toll protein, which was originally identified as a receptor that plays a central role in the establishment of the embryonic dorsal-ventral pattern in fly embryos [1]. Growing evidence suggests that TLRs have a critical role in the recognition of bacterial lipoproteins, major components of bacterial cell walls. Their signaling pathway is known to activate target genes such as nuclear factor-κB (NF-κB) and cytokines that are involved in inflammation and immune responses [2-4]. In 1997, Medzhitov et al. demonstrated that a Toll-like receptor now known as TLR4 could, when artificially ligated using antibodies, induce the activation of certain genes necessary for initiating an adaptive immune response [5]. When activated, TLRs recruit adapter molecules within the cytoplasm of cells in order to propagate a signal. Four adapter molecules are known to be involved in signaling. These proteins are known as myeloid differentiation primary response gene-88 (MyD88), Mal/TIRAP (Mal is also called TIR domain-containing adaptor protein [TIRAP]), TIR domain-containing adaptor-inducing interferon-β (TRIF) and TRIF-related adaptor molecule (TRAM) interacting with TLRs in response to ligand stimulation. When engaged by gram-negative lipopolysaccharide (LPS), this complex transduces a signal detected by MyD88, which is passed onward by a cascade of the receptor-associated kinase (IRAKs), receptor-associated factor 6 (TRAF6), and NF-kappa B inducing kinase (NIK), resulting in activation of NFκB which amplifies the signal, and ultimately leads to the induction or suppression of genes that orchestrate the inflammatory response [2,4].

Recent studies have shown that there is an overexpression of TLR2 and TLR4 in intestinal epithelial cells which is correlated with the severity of mucosal damage, together with an increase of apoptotic cells and markedly impaired proliferation [5]. The increased expression of TLRs was found in the intestinal tissues during experimental necrotizing enterocolitis (NEC) which is a well known pediatric intestinal inflammatory disease [6]. TLRs when activated by bacterial ligands signal intracellularly to up-regulate the expression of various cytokines in the intestine, including pro-(IL-1β, IL-6, IL-8) and anti-inflammatory cytokines (IL-10) in human and different NEC models [7,8].

Oral and gastrointestinal mucositises are common complications of chemotherapy, in particular with drugs affecting DNA synthesis (S-phase-specific agents such as fluorouracil, methotrexate, and cytarabine). Mucositis occurs in 40% of patients after standard dose chemotherapy, and in 100% of patients undergoing high dose chemotherapy and stem cell or bone marrow transplantation and contributes not only to the morbidity of treatment but also to its cost [9,10]. The pathogenesis of chemotherapy induced gastrointestinal mucositis has been described by Sonis et al. [11] and includes five phases: initiation by chemotherapy, up-regulation and generation of messenger signals, signaling by pro-inflammatory cytokines and amplification of mucosal injury, ulceration of the mucosa and finally, healing. The initial stages of inflammation in mucositis include increased pro-inflammatory cytokine levels, which act as a homing marker for inflammatory immune cells in the submucosa.

Glutamine is a nonessential amino acid which is beneficial in the prevention of infectious morbidity and mortality in seriously ill patients, in part due to its ability to maintain the integrity of intestinal mucosal epithelium. Extensive studies in various experimental models have established that glutamine is an essential respiratory substrate for cells in the small intestinal mucosa, accounting for over one-third of the total CO_2_ produced in the small intestine [12]. While in the large bowel the short chain fatty acid butyrate is the preferred fuel for the colonic mucosa, many experimental studies have indicated that glutamine is the preferred fuel source for the small intestine [13,14]. In addition, glutamine exerts a positive effect on the gut-associated lymphoid tissue and enhances gut barrier function. We have demonstrated recently that administration of enteral glutamine prevents intestinal mucosal damage and accelerates intestinal recovery following methotrexate-induced mucositis in a rat [15]. In this study, we showed that treatment with glutamine attenuates alterations in TLR-4, MyD88 and TRAF6 intestinal expression during LPS endotoxemia in a rat [16].

In the current study we hypothesized that TLR signaling may be involved in the development of MTX-induced intestinal mucositis and that treatment with glutamine might reduce the alterations in this pathway.

## Methods

### Animals

This experiment and animal care were conducted in compliance with the guidelines established by the “Guide for the Care and Use of Laboratory Animals”, Rappaport Faculty of Medicine, Technion (Haifa, Israel). Male Sprague–Dawley rats (250-300 g) were used in this study. Animals were housed in wire-bottomed cages and were acclimatized at 21°C on 12:12-h light–dark cycle for a minimum of five days before the experiment. The rats were allowed access to water and chow ad libitum.

### Experimental design

Animals were randomly assigned to one of four experimental groups of 8 rats each: 1) control rats (Group A) underwent IP injection of sterile saline (CONTR); 2) Group B (CONTR-GLN) animals were treated with oral glutamine given in drinking water (2%) 48 hours before and 72 hours following vehicle injection; 3) Group C rats (MTX) were treated with a single IP injection of MTX (20 mg/kg); and 4) Group D (MTX-GLN) were pre-treated with oral glutamine given similar to group B rats, 48 hours before and 72 hours after MTX injection.

### Intestinal mucosal parameters

Three days following MTX or vehicle injection, animals were anesthetized with IP sodium pentobarbital (75 mg/kg) and were sacrificed by open pneumothorax. The small bowel was excised quickly, washed with cold isotonic saline and divided into two segments: proximal jejunum and terminal ileum. Each segment was weighed and cut longitudinally. Mucosa was scraped using a glass slide, collected and weighed. Total RNA, DNA and protein from the jejunum and ileum were extracted sequentially as described by Chomczynski [17]. Quantitization of DNA was performed by spectrophotometry using A260 value (one A260 unit equals 50 μg of double-stranded DNA) and calculated as μg/cm bowel length/100 g body weight.

### Histological examination

Tissue samples were removed from the jejunum and ileum and were immediately fixed in 4% neutral-buffered formalin. The samples were then embedded in paraffin and sectioned. Deparaffinized 5 μm sections were stained with haematoxylin and eosin. The mucosal damage of the small bowel was graded using an intestinal injury score as described by Kesik et al. [18]. The following parameters were investigated in the jejunum and ileum: (1) degeneration of surface and crypt epithelium, (2) degeneration of villus structure and vacuolization in the surface epithelium, and (3) inflammatory cell infiltration and bleeding and edema in the lamina propria. For each parameter, a score was given using a semiquantitative scale as follows: 0 = none, 1 = mild, 2 = moderate, 3 = severe, giving a maximum possible score of 9 for each intestinal region.

The villus height and crypt depth for each specimen were measured using an objective mounted micrometer (100 × magnification) and an optical microscope (10 × 100 magnification). Villus height and crypt depth data were taken from eight rats, and each measurement consisted of the mean of ten villi and crypts.

### Enterocyte proliferation and apoptosis

Crypt cell proliferation was assessed using 5-bromodeoxyuridine (5-BrdU). Standard BrdU labeling reagent (Zymed Laboratories, Inc, San Francisco, CA) was injected intraperitoneally at a concentration of 1 ml/100 g body weight 2 hours before sacrifice. Tissue slices (5 μm) were stained with a biotinylated monoclonal anti-BrdU antibody system provided in a kit form (Zymed Laboratories, Inc, San Francisco, CA). An index of proliferation was determined as the ratio of crypt cells staining positively for BrdU per 10 crypts.

Additional 5 μm thick sections were prepared to establish the degree of enterocyte apoptosis. Immunohistochemistry for Caspase-3 (Caspase-3 cleaved concentrated polyclonal antibody; dilution 1:100; Biocare Medical, Walnut Greek, CA) was performed for identification of apoptotic cells using a combination of the streptovidin-biotin-peroxidase method and microwave antigen retrieval on formalin-fixed, paraffin-embedded tissues according to the manufacturer’s protocols. The apoptotic index (AI) was defined as the number of apoptotic cells per 10 villi.

A qualified pathologist blinded as to the source of intestinal tissue performed all measurements.

### TLR4, MyD88 and TRAF6 mRNA expression (Real time PCR analysis)

Expression of TLR4, MyD88 and TRAF6 mRNA levels was determined by quantitative real-time PCR (7500 Real-Time PCR System, Applied Biosystems, USA) on cDNA samples using Cyber Green Master Mix (ROVALAB, Germany) with the exception of template and primers. Primers for Rattus norvegicus TLR4, MyD88 and TRAF6 were synthesized by Syntezza Bioscience ltd.Israel, and 18 s rRNA Control kit from Eurogentec, EGT Group.

### Western blot analysis

Tissue was homogenized in RIPA lysis buffer containing 50 mM Tris–HCl (pH 7.4), 150 mM NaCl, 1% NP-40, 2 mM EDTA, supplemented with a cocktail of protease and phosphatase inhibitors. Protein concentrations were determined by Bradford reagent according to the manufacturer’s instructions. Samples containing equal amounts of total protein (30 μg) were resolved by SDS-PAGE under reducing conditions. After electrophoresis, proteins were transferred to a PVDF membrane and probed with various primary antibodies to anti-TLR-4 antibody (1:100 dilution), anti-MyD88 antibody (1:200 dilution) and anti-ERK antibody (1:1000 dilution, sc-56899). Horseradish peroxidase-conjugated secondary antibody was purchased from Jackson ImmunoResearch Laboratories Inc. (West Grove, PA) and an enhanced chemiluminescent substrate from Biological Industries (Kibbutz Beth HaEmek, Israel). The optical density of the specific protein bands was quantified by using a densitometer (Vilber Lourmat, Lyons, France).

### Immunohistochemistry for TLR-4 and TRAF6 expression

Immunohistochemistry for TLR-4 and TRAF6 (TLR-4 polyclonal antibody; dilution 1:100, Santa Cruz,CA and TRAF6 polyclonal antibody; dilution 1:100, Santa Cruz,CA) was performed to identify TLR-4 and TRAF6 expression, using a combination of streptovidin-biotin-peroxidase method according to manufacturers’ protocols. The paraffin-embedded sections were de-waxed and rehydrated with xylene and graded alcohol. Tissue sections were microwave-pretreated in 10 mM EDTA buffer and incubated with an endogenous peroxidase (3%) in methanol for 10 minutes. After incubation with blocking solution at room temperature for 10 minutes, the sections were then incubated with TLR-4 and TRAF6 concentrated polyclonal antibody for 60 minutes and second human-absorbed, biotinylated, affinity-purified antibody for 20 minutes. TLR-4 and TRAF6 receptor positive color development was obtained by incubating the sections with DAB (Deoxyaminobenzidine) substrate (Zymed Laboratories). A qualified pathologist blinded as to the source of the intestinal tissue performed all measurements.

### Statistical analysis

The data are expressed as the mean ± SEM. A one-way ANOVA for comparison, followed by Tukey’s test for pair-wise comparison was used for statistical analysis. Prism software was used (GraphPad Software, Inc., San Diego, CA) and statistical significance was defined as *P* < 0.05.

## Results

### Intestinal mucosal parameters

MTX-induced intestinal damage (Group C) resulted in a significant decrease in bowel weight in jejunum (10%, p < 0.05) and ileum (12%, p < 0.05), mucosal weight in jejunum (29%, p < 0.05), and ileum (20%), mucosal DNA in jejunum (three-fold decrease, p < 0.05), and ileum (30%, p < 0.05), and mucosal protein in jejunum (18%, p < 0.05), and ileum (22%, p < 0.05) compared to control animals (Group A) (Table 1). Following oral glutamine administration, MTX-rats (Group D) demonstrated a significant increase in ileal bowel weight (11%, p < 0.05), jejunal (19%, p < 0.05) and ileal (27%, p < 0.05) mucosal weights, and jejunal (23%, p < 0.05) and ileal (29%, p < 0.05) protein content compared to MTX-untreated animals (Group C).

**Table 1:** Effect of MTX and glutamine on intestinal mucosal parameters

**Paramaters/Groups**	**CONTR**	**CONTR-GLN**	**MTX**	**MTX-GLN**
Bowel weight (mg/cm/100 g BW)				
Jejunum	22.6 ± 0.9	21.3 ± 0.5	20.3 ± 0.4*	19.7 ± 1.3
Ileum	20.0 ± 0.9	19.0 ± 0.8	18.3 ± 0.8*	20.3 ± 1.1†
Mucosal weight (mg/cm/100 g BW)				
Jejunum	9.7 ± 0.6	10.1 ± 0.6	6.9 ± 0.5*	8.2 ± 0.6*†
Ileum	9.0 ± 0.5	8.4 ± 0.3	7.2 ± 0.2*	9.1 ± 0.7†
Mucosal DNA (μg/cm/100 g BW)				
Jejunum	4.5 ± 0.4	4.2 ± 1.4	1.6 ± 0.4*	1.6 ± 0.2*
Ileum	7.4 ± 0.8	5.5 ± 1.5	5.2 ± 0.5*	5.2 ± 0.6*
Mucosal protein (μg/cm/100 g BW)				
Jejunum	160 ± 13	184 ± 30	132 ± 7*	163 ± 12†
Ileum	166 ± 8	174 ± 21	130 ± 11*	168 ± 16†

CONTR-control, GLN-glutamine, MTX-methotrexate.

*P < 0.05 vs control, †P < 0.05 MTX-GLN vs MTX.

### Histological changes

**Figure 1:**
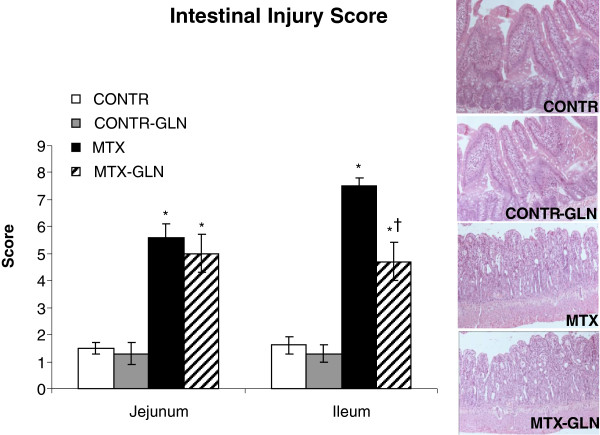
**Effects of MTX and enteral glutamine on intestinal injury score.** The following parameters were investigated: (1) degeneration of surface and crypt epithelium, (2) degeneration of villus structure and vacuolization in the surface epithelium (3) inflammatory cell infiltration and bleeding and edema in the lamina propria. For each criterion, a score was given using a semiquantitative scale as follows: 0 = none, 1 = mild, 2 = moderate, 3 = severe, giving a maximum possible score of 9 for each segment. Values are mean ± SEM. CONTR-control, MTX-methotrexate, GLN-glutamine. *P < 0.05 MTX and MTX-GLN vs CONTR rats. †P < 0.05 MTX-GLN vs MTX rats.

Administration of GLN in control animals (Group B) had no significant effect on the semi-quantitative damage score compared to control rats (Group A) (Figure 1). MTX-animals histologically exhibited a significant loss of crypt architecture and signs of crypt remodeling, severe villous epithelial atrophy, degeneration and shortening of the villus length, and polymorphonuclear leukocyte infiltration in the lamina propria. Histological damage was initially assessed in jejunum and ileum by the semi-quantitative score, which included three criteria that were markedly affected by methotrexate. Intestinal damage as a result of methotrexate administration was greatest in the distal ileum (7.5 ± 0.3 vs 1.6 ± 0.3, p < 0.001) and was less significant in the proximal jejunum (5.6 ± 0.5 vs 1.5 ± 0.02, p < 0.001). Treatment of MTX rats with enteral GLN (Group D) resulted in a significant decrease in the intestinal damage score in the distal ileum (4.7 ± 0.7 vs 7.5 ± 0.3, p < 0.001) compared to MTX-animals (Group C).

MTX-rats (Group C) showed a significant decrease (vs control animals, Group A) in villus height in jejunum (two-fold decrease, p < 0.05) and ileum (37% decrease, p < 0.05) as well as crypt depth in jejunum (43% decrease, p < 0.05) and ileum (34% decrease, p < 0.05) compared to control animals (Group A) (Table 2). MTX-GLN rats (Group D) demonstrated a significantly greater villus height in jejunum (14% increase, p < 0.05) and ileum (32% increase, p < 0.05), as well as crypt depth in jejunum (34% increase, p < 0.05) and ileum (33% increase, p < 0.05) compared to MTX-untreated animals (Group C).

**Table 2:** Effect of MTX and glutamine on microscopic intestinal parameters, cell proliferation and apoptosis

**Parameters/Groups**	**CONTR**	**CONTR-GLN**	**MTX**	**MTX-GLN**
Villus height (μm)				
Jejunum	461 ± 16	460 ± 40	246 ± 11*	281 ± 14*†
Ileum	304 ± 26	294 ± 30	192 ± 17*	255 ± 11*†
Crypt depth (μm)				
Jejunum	184 ± 7	148 ± 18	105 ± 8*	138 ± 10*†
Ileum	157 ± 7	131 ± 9	102 ± 3*	136 ± 13*†
Cell proliferation (BrdU positive cells/10 crypts)				
Jejunum	158 ± 9	163 ± 13	132 ± 4*	147 ± 5†
Ileum	165 ± 14	189 ± 25	129 ± 4*	152 ± 5†
Cell apoptosis (apoptotic cells/10 villi)				
Jejunum	0.7 ± 0.2	0.9 ± 0.26	1.45 ± 0.23*	1.23 ± 0.23
Ileum	0.3 ± 0.05	0.5 ± 0.14	1.44 ± 0.13*	0.85 ± 0.26*†

CONTR-control, GLN-glutamine, MTX-methotrexate.

*P < 0.05 vs control, †P < 0.05 MTX-GLN vs MTX.

### Enterocyte proliferation and apoptosis

MTX rats (Group B) demonstrated a significant decrease in the rate of enterocyte proliferation in both jejunum (16% decrease, p < 0.01) and ileum (21% decrease, p < 0.05) and a concomitant increase in the apoptotic index in jejunum (two-fold, p < 0.05) and ileum (five-fold, p < 0.05) compared to control animals (Table 2). Treatment with oral glutamine (Group D) led to a significant increase in the enterocyte proliferation rate in jejunum (11% increase, p < 0.05) and ileum (18% increase, p = 0.001), and a concomitant decrease in the enterocyte apoptosis in ileum (42% decrease, p < 0.05) compared to MTX-animals (Group C).

### TLR-4, MyD88 and TRAF6 mRNA expression

**Figure 2:**
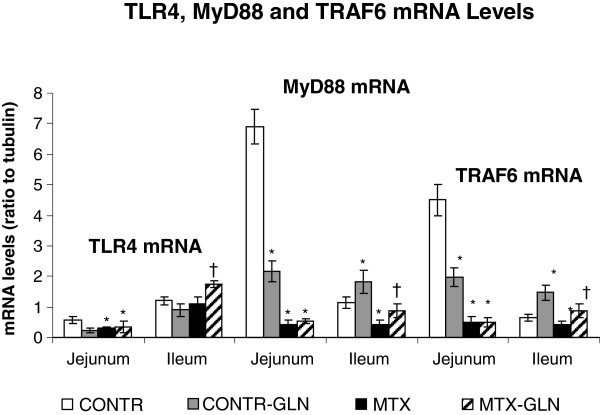
**Evolution of mRNA expression levels of TLR4, MYD 88 and TRAF6 from the ileum of MTX and glutamine treated rats over 72 h.** RNA was isolated from the ileum of rats subjected to MTX alone or to glutamine supplementation for 72 h. Real time PCR analysis was performed to determine the expression level of TLR4, MYD 88 and TRAF6 mRNA comparing control, MTX with glutamine-fed rats. Values are mean ± SEM. CONTR-control, MTX-methotrexate, GLN-glutamine. *P < 0.05 MTX and MTX-GLN vs CONTR rats. †P < 0.05 MTX-GLN vs MTX rats.

MTX rats (Group C) showed a significant decrease in TLR-4 mRNA expression in jejunum (two-fold decrease, p < 0.05), MyD88 mRNA expression in jejunum (15-fold decrease, p < 0.05) and ileum (four-fold decrease, p < 0.05), and TRAF6 mRNA expression in jejunum (10-fold decrease, p < 0.05) and ileum (37% decrease, p < 0.05) compared to control animals (Group A) (Figure 2). Treatment of MTX rats with glutamine (Group D) resulted in a significant increase in ileal TLR-4 mRNA (60% increase, p < 0.05), ileal MyD88 mRNA (two-fold decrease, p < 0.05) and ileal TRAF 6 mRNA expression (two-fold decrease, p < 0.05) compared to MTX-rats (Group C). MTX-GLN rats also showed a trend toward an increase in TLR4 and MyD88 mRNA levels in jejunum; however, this trend did not achieve statistical significance.

### Expression of TLR-4 and MyD88 proteins

**Figure 3:**
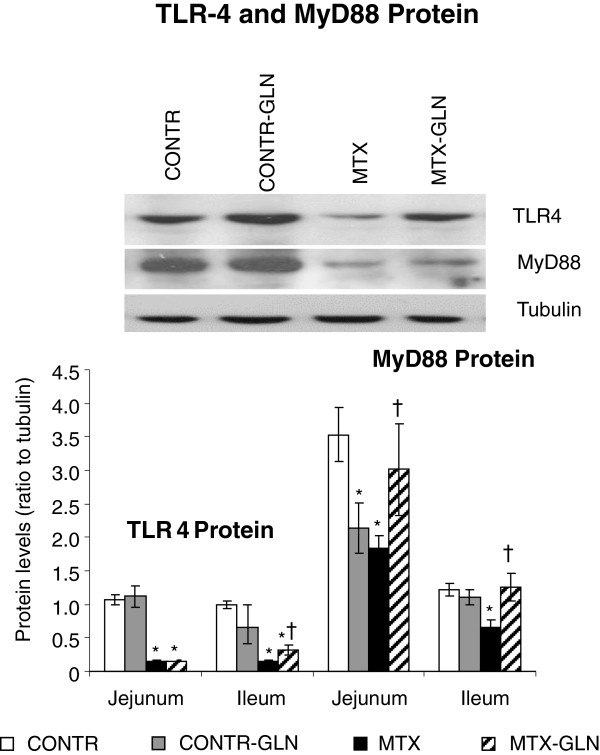
**Expression of TLR4 and MyD 88 proteins in the jejunum and ileum.** Tissue lysates were prepared for Western blot analysis and 50 μg of total protein were loaded and immunoblotted with anti- TLR4 and MyD 88 and TRAF6 antibodies, with tubulin as a loading control. *Top*: a typical blot. *Bottom*: relative densitometric values are means ± SE. MTX rats demonstrated a significant decrease in TLR-4 and MyD88 expression in jejunum and ileum compared to control animals. Protein expression of TLR-4 and MyD88 increased following glutamine administration. CONTR-control, MTX-methotrexate, GLN-glutamine. *P < 0.05 MTX and MTX-GLN vs CONTR rats. †P < 0.05 MTX-GLN vs MTX rats.

Western blot analysis confirmed the changes in TLR-4 and MyD88 (Figure 3). A significant decrease in TLR-4 protein expression in jejunum (7-fold decrease, p < 0.05) and ileum (7-fold decrease, p < 0.05) and MyD88 protein expression in jejunum (two-fold decrease, p < 0.05) and ileum (30% decrease, p < 0.05) was found in MTX rats (Group C) compared to control animals (Group A). Treatment of MTX -rats with glutamine (Group D) resulted in significant increase in TLR-4 protein in ileum (two-fold, p < 0.05) and MyD88 protein in jejunum (90%, p < 0.05) and ileum (two-fold, p < 0.05) compared to MTX animals (Group C).

### Immunohistochemistry for TLR-4 and TRAF6 expression

**Figure 4:**
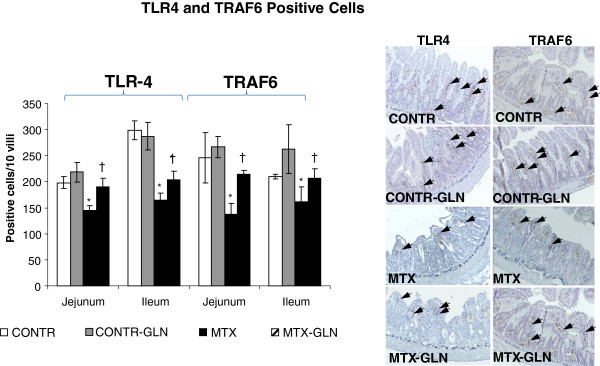
**Immunohistochemical localization of TLR4 and TRAF6 in jejunal and ileal tissues.** Magnification is 200. TLR4 and TRAF 6 were expressed on intraepithelial lymphocytes and lymphocytes in submucosa. In addition weak positive staining was observed in the crypt region. MTX-induced damage was associated with decreased TLR4 and TRAF6 staining. The number of TLR4 and TRAF6 positive cells increased significantly following glutamine administration. CONTR-control, MTX-methotrexate, GLN-glutamine. *P < 0.05 MTX and MTX-GLN vs CONTR rats. †P < 0.05 MTX-GLN vs MTX rats.

Expression of TLR4 and TRAF6 (Figure 4) in rat intestinal tissues was also investigated in control, CONTR-GLN, MTX and MTX-GLN animals using immunohistochemistry. In control and CONTR-GLN rat intestinal tissues, both TLR4 and TRAF6 expression stained with a stronger intensity along the entire villus-crypt axis. TLR4 and TRAF 6 were expressed on intraepithelial lymphocytes and lymphocytes in the submucosa. In addition, weak positive staining was observed in the crypt region. Treatment of control animals with glutamine (Group B) resulted in a trend toward increase in the number of TLR-4 positive cells in jejunum as well as in the number of TRAF-6 positive cells in jejunum and ileum compared to control animals; however, this trend was not statistically significant. MTX-induced intestinal damage (Group C) was associated with a significant decrease in TLR-4 staining in jejunum (145 ± 17 vs 198 ± 11 positive cells/10 villi, p < 0.05) and ileum (164 ± 17 vs 299 ± 11 positive cells/10 villi, p < 0.05) as well as in TRAF-6 staining in jejunum (two-fold decrease, p < 0.05) and ileum (161 ± 18 vs 210 ± 5 positive cells/10 villi, p < 0.05) compared to control animals (Group A). Treatment of MTX animals with glutamine diminished this inhibitory effect of MTX on TLR-4 and TRAF-6 expression. MTX-GLN animals (Group D) demonstrated a significant increase in TLR-4 staining in jejunum (191 ± 10 vs 145 ± 17 positive cells/10 villi, p < 0.05) and ileum (204 ± 13 vs 164 ± 17 positive cells/10 villi, p < 0.05) as well as in TRAF-6 staining in jejunum (215 ± 20 vs 138 ± 7 positive cells/10 villi, p < 0.05) and ileum (207 ± 29 vs 161 ± 18 positive cells/10 villi, p < 0.05) compared to MTX animals (Group C).

## Discussion

Mucositis is an important side effect of methotrexate therapy for which there is no definitive prophylaxis or treatment. This is due in part to the lack of understanding of its pathogenesis and its impact on intestinal structure and function. Over the last decades, significant progress has been made in understanding the underlying mechanisms of mucositis development. The pathobiology of mucositis is complex and includes up-regulation of a range of stress response cytokines and subsequent activation of mitogen activated protein kinase (MAPK) signaling, nuclear factor κB (NFκB) signalling, Fos/Jun signalling and Wnt signaling [19,20]. The current hypothesis for the development of mucositis includes five biological phases, namely: *initiation*, which encompasses the primary damage occurring following administration of cytotoxic chemotherapy; message *generation*, involving the up-regulation of transcription factors including NF*κ*B, MAPK, matrix metalloproteinases and subsequent activation of cytokine and stress response genes; *signalling and amplification* producing proteins such as tumour necrosis factor, interleukin-1*β* and interleukin-6 which cause direct tissue damage and provide positive feedback to amplify the process; *ulceration*, which resuls in painful ulcers, bacterial infiltration and an influx of macrophages and other inflammatory cells and finally *healing*, which spontaneously occurs upon cessation of chemotherapy [11].

Possible mediators of mucositis which have not been well studied are related to toll-like receptor signaling. TLR signaling has been shown to function in several of the pathways which mediate mucositis development and tissue injury in general. Therefore, TLRs deserve careful consideration as possible mediators of chemotherapy-induced mucositis. Toll-like receptors are an ancient conserved receptor family that regulate antimicrobial host defense in plants, invertebrates, and mammals [21]. Individual TLRs recognize distinct pathogen-associated molecular patterns (PAMPs) that have been evolutionarily conserved in specific classes of microbes. Interaction of PAMPs with TLRs triggers a complex signaling pathway that leads to the activation of the immune system. There are more than 10 identified members of the TLR family, with well-defined specificities to various components of bacteria, viruses, and fungi [22]. The best characterized member of this family is toll-like receptor 4 (TLR-4), the receptor for lipopolysaccharide, which is the best known and first discovered bacterial cell wall component that can elicit cellular responses. TLR-4 is responsible for the recognition of bacterial endotoxin or LPS and for initiation of the gram-negative bacillary septic shock syndrome [23,24]. Interaction between LPS and TLR-4 leads to formation of an LPS signaling complex consisting of surface molecules, such as CD14 and MD2, as well as intracellular adaptor molecules, including myeloid differentiation primary response gene 88 (MyD88), TNF-α receptor association factor 6 (TRAF6), and activation of transcription factors, such as nuclear factor κB (NFκB), which then induce the activation of the inflammatory genes, such as tumor necrosis factor-α, interleukin (IL)-1, IL-6, and IL-8 [5].

Epithelial cells of the gastrointestinal tract (GIT) undergo continuous renewal. The process of cell turnover is tightly regulated by mucosal as well as submucosal signalling in order to maintain homeostasis and to compensate for disturbances which may occur in the GIT. The distribution and expression of TLRs in the basement membrane plays a predominant role in regulating epithelial cell kinetics. Therefore, it is important that TLRs levels are continuously regulated to achieve a balance in tissue degradation and fibrogenesis. Furthermore, a skewed level of tissue TLRs has been implicated in the development of acute and chronic intestinal diseases.

The TLR signaling following chemotherapy has not received a great deal of attention. Previous studies have shown a role for ROS and the pro-inflammatory cytokines TNF and IL-1*β* in the message generation phase of mucositis [25] and in the induction of TLR signaling [26], separately, with no studies documenting a direct relationship between TLR signaling and the development of intestinal mucositis.

Glutamine is a non-essential amino acid which plays an important role in many physiologic and biologic processes. Growing evidence suggests that glutamine is an important nutrient for rapidly dividing cells such as those from the immune system and the gut [12]. We have shown that treatment with oral glutamine prevents mucosal injury and improves intestinal recovery following MTX- injury in the rat [14]. In this other experiment, we demonstrated a correlation between gut trophic effects of glutamine and its stimulating effect on TLR signaling during lipopolysaccharide endotoxemia in a rat [15].

We hypothesized in the present study that this TLR4 signaling is involved during MTX-induced mucositis and that glutamine could prevent intestinal mucosal injury or/and improve intestinal recovery by affecting this pathway. Our data has shown that a single dose of MTX can cause a severe mucosal injury in the small bowel. This is evident from the increased intestinal injury score. Histologically, MTX-animals exhibited degeneration and shortening of the villus length, severe villous epithelial atrophy, significant loss of crypt architecture, signs of crypt remodeling, and polymorphonuclear leukocyte infiltration in the lamina propria. In addition, MTX rats showed intestinal mucosal hypoplasia. The observed decreased bowel and mucosal weight, decreased mucosal DNA, decreased villus height and crypt depth support this conclusion. As a folic acid analogue, the action of MTX primarily is to inhibit DNA synthesis by binding to the enzyme dihydrofolate reductase. This leads to an inhibition of proliferation in the crypts of the small intestine. Our data support this concept. Mucosal DNA content as well as enterocyte proliferation index decreased significantly in both jejunum and ileum following MTX administration. Cell death via apoptosis increased significantly in intestinal mucosa after MTX administration. The loss of small intestinal epithelial cells leading to villous atrophy caused by MTX has been well described and has been appreciated since the 1970s [27,28].

We found that administration of MTX was associated with down-regulation of TLR-4 Myd88 and TRAF-6 mRNA expression in the jejunal and ileal mucosa. The elevation of TLR-4 and Myd88 mRNA expression in the ileum was found to be statistically significant. Changes in protein levels were in agreement with mRNA expression. Both TLR-4 and Myd88 protein levels were significantly down-regulated in MTX-treated rats compared to control animals. The down-regulation expression of TLR-4, Myd88 and TRAF-6 mRNA and protein expression were poorly correlated with severe mucositis, which leads to induction of inflammatory cytokines such tumor necrosis factor- α, IL-1, and IL-6; and, therefore, cannot explain the developed widespread intestinal mucosal injury. Previous imunohistochemical techniques have shown that TLR4 is expressed at low levels by intraepithelial cells in normal human colon tissues and predominantly in the crypt epithelial cells [29,30]. In the current study, both TLR4 and TRAF6 expressions stained with a stronger intensity along the entire villus-crypt axis and were expressed on intraepithelial lymphocytes and lymphocytes in submucosa. In addition, weak positive staining was observed in the crypt region. MTX-induced intestinal damage was associated with a significant decrease in TLR-4 and TRAF-6 staining in jejunum and ileum compared to control animals. These changes were in agreement with changes in mRNA and protein levels. Since TLR4 signaling is pro-inflammatory as well as administration of MTX causes inflammatory response, it should be emphasized that the down-regulation of components of the TLR signaling cascade by MTX is presumably compensatory. Such compensation mechanisms are common, activated receptors mediate their own downregulation to limit/stop the response to the stimulus.

We demonstrated previously the beneficial effects of glutamine on methotrexate induced mucositis [15]; however, the exact mechanism of this positive effect remains unclear. Consistent with these data, in the current study enteral glutamine produced various beneficial effects. The results of this study have shown that glutamine given during 72 hours to control animals did not significantly change intestinal mucosal parameters. However, pretreatment with glutamine protected the intestinal mucosa from damage caused by MTX. 80% of rats showed a significant decrease in intestinal mucosal injury grade compared to MTX animals, suggesting lesser degree of intestinal damage. In addition, exposure to enteral glutamine accelerated intestinal mucosal repair and enhanced enterocyte turnover. This is evident from the significant increase in bowel and mucosal weight, increased DNA content and increased villus height and crypt depth in this model. Treatment with glutamine led to a rapid increase in the uptake of bromdioxyuridine (BrdU) and the size of the proliferative compartment in the crypts. After exposure to glutamine supplementation, an abnormally “rich” supply of glutamine entering the small intestine might directly stimulate mucosal hyperplasia. The epithelial cells lining the intestinal canal probably use this amino acid for their own nutrition. The increased absorption of glutamine from the lumen may also stimulate the release and circulation of enteric hormones which have trophic effects on small bowel mucosa. An increased cell proliferation rate was accompanied by increase in villus height, suggesting an increased absorptive surface area. Increased cell proliferation following glutamine administration was accompanied by decreased cell apoptosis. The specific mechanism for the apparent anti-apoptotic effects of glutamine has not yet been elucidated fully. Glutamine may attenuate the enterocyte injury that precedes apoptosis, possibly as a membrane stabilizer or as a free-radical scavenger, thereby preventing or delaying the initiation of the apoptotic cascade. Alternatively, glutamine may act intracellularly to alter the expression of genes related to apoptosis or interfere with specific caspase functions.

Treatment with glutamine in the current study attenuated the inhibitory effect of MTX on TLR-4 signaling. MTX-GLN rats demonstrated a significant increase in ileal TLR-4 mRNA, MyD88 mRNA and TRAF 6 mRNA expression compared to MTX-rats, which was correlated with a trend toward increase in TLR-4 and MyD88 protein, and with the increased number of TLR-4 and TRAF-6 positive cells (by immunohistochemistry). These findings suggest that glutamine stimulates TLR signaling pathways, which is in agreement with other experiments. It has been reported that TLR2 and TLR4 expression is up-regulated under inflammatory conditions [31], which could provide a mechanism for certain inflammatory diseases of the intestine. Indeed, there is a report of increased TLR4 expression in the intestinal epithelium of patients with Crohn’s disease and ulcerative colitis [32]. Whether TLR4 expression is a cause or a result of the disease is unclear, but it does underscore the importance of the gastrointestinal epithelium in preventing excessive and uncontrolled inflammation. Current data suggests that TLRs are differentially expressed in intestinal mucosa during chemotherapy-induced mucositis on both leukocytes and mucosal epithelial cells while serving to modulate leukocyte-epithelial interactions. It should be emphasized that aberrant TLR-4 expression may play an important role in the loss of tolerance to the enteric bacteria during chemotherapy-induced mucositis. Glutamate, by increasing expression of components of the TLR signaling pathway, would be expected to amplify the inflammatory response and further increase damage tissue. That it has the opposite effect suggests that glutamate may be acting independently of TLR signaling.

The mechanism of the positive effect of glutamine on gut barrier function is poorly understood. Recent evidence suggests that glutamine prevents MTX-induced gut barrier disruption by regulating occludin and claudin-1 probably through erk and NF-κB pathways [33]. Since TLR4-MyD88/Mal-NF-kB signaling axis plays an important role in gut barrier function, further experiments are required to understand crosstalk between TLR-signaling and NF-kB-signaling during chemotherapy-induced mucositis.

In summary, methotrexate inhibits TLR-4 signaling pathways. Glutamine improves intestinal recovery and attenuates this inhibitory effect. The positive effect of glutamine on intestinal structure and gut barrier function in intestinal mucositis may be considered as a mechanism by which immunonutrition helps in the recovery of oncologic patients receiving chemotherapy.

## Competing interests

The authors declare that they have no competing interests.

## Authors’ contributions

This work was supported by a research grant from Israeli Ministry of Health No 3/6164 from 01/06/10 and Hedson Fund for Medical Research, Technion, Haifa, Israel. IS, YP and JP carried out the animal study, performed immunohistochemistry, and were responsible for data collection. JB was responsible for histological analysis. IS, AGC, JGM and DB participated in the sequence alignment, carried out the statistical analysis and drafted the manuscript. All authors read and approved the final manuscript.

## Acknowledgement

This work was supported by a research grant from Israeli Ministry of Health No 3/6164 from 01/06/10 and Hedson Fund for Medical Research.
